# Building resilience in German primary care practices: a qualitative study

**DOI:** 10.1186/s12875-022-01834-4

**Published:** 2022-09-02

**Authors:** Nicola Litke, Aline Weis, Jan Koetsenruijter, Valeska Fehrer, Martina Koeppen, Stephanie Kuemmel, Joachim Szecsenyi, Michel Wensing

**Affiliations:** 1 Department of General Practice and Health Services Research, Heidelberg University Hospital, Im Neuenheimer Feld 130.3, Marsilius Arkaden, Turm West, D-69120 Heidelberg, Germany; 2 aQua Institute for Applied Quality Improvement and Research in Health Care GmbH, Göttingen, Germany

**Keywords:** Primary care practice, Primary care, Crisis, Resilience, Preparedness, Climate change

## Abstract

**Background:**

In recent years, healthcare has faced many different crises around the world such as HIV-, Ebola- or H1N1-outbrakes, opioid addiction, natural disasters and terrorism attacks). In particular, the current pandemic of Covid-19 has challenged the resilience of health systems. In many healthcare systems, primary care practices play a crucial role in the management of crises as they are often the first point of contact and main health care provider for patients. Therefore, this study explored which situations are perceived as crises by primary care practice teams and potential strategies for crisis management.

**Methods:**

A qualitative observational study was conducted. Data were collected in interviews and focus groups with experts from primary care practices and stakeholders focusing on primary care practices in Germany such as physicians, medical assistants, practice managers, quality managers, hygiene managers and institutions on health system level (politics, research and health insurance). All interviews and focus groups were audio-recorded and transcribed verbatim. A qualitative content analysis was performed using a rapid qualitative analysis approach first, followed by a thematic analysis.

**Results:**

Two focus groups and 26 interviews including 40 participating experts were conducted. Many different situations were perceived as crises, varying from issues in the practice organization to problems on health system level and international disasters. Distinct aspects associated with the perception of a crisis situation by interviewees were the presence of emotional reactions, a need for organizational changes and a lack of necessary resources. A broad spectrum of possible strategies was discussed that could help to cope with or even prevent the emergence of an actual crisis. In particular, strengthening communication within practice teams and resilience among employees was perceived to be fundamental for improving responses to crises or preventing them.

**Conclusions:**

The study provides perspectives of primary health care workers on crises in health, that could inform health policy regarding prevention and management of future crises in primary care facilities.

**Supplementary Information:**

The online version contains supplementary material available at 10.1186/s12875-022-01834-4.

## Background

In recent years, health care faced many crises such as HIV, Ebola, H1N1, opioid addiction, natural disasters and terrorism attacks. Particularly the current pandemic of Covid-19 has challenged the resilience of health systems globally. Furthermore, future crises related to the impacts of climate change, epidemics, wars and pandemics have been predicted [1]. Building resilience to face those potentially upcoming challenges in health care is therefore crucial.

Crises have also been described as “disasters”, “shocks” and ‘’surges’’ [2–4]. These terms all refer to a sudden increase in the incidence of health problems, which has major impact on the need for health care. Crises differ from other challenges, such as the ageing population and the increase of multimorbid patients by their attribution to specific events, although a clear differentiation can be difficult. In the literature, there are many different definitions of resilience of health systems. Most of them have in common that they describe it “as the degree of change a system can undergo while maintaining its functionality” [5]. By managing past crises, different strategies have been developed and various articles stating lessons learned have been published [5–8]. Most contributions on this topic take a health system and population health perspective, while less is known about the experiences and responses of healthcare providers. For instance, the resilience of healthcare workers in the current Covid-19 pandemic seems to be affected dramatically [9, 10]. In Germany, primary care practices are central in the management of the Covid-19 pandemic as they are often first and only point of contact with health care for infectious patients [11].

To prepare for future crises and build resilience in primary care, the project “RESILARE – building resilience of primary care practices by developing and evaluating quality indicators” was initiated. Within this project, quality indicators will be developed that aim to measure crisis resilience of primary care practices in Germany and point out approaches to support practices and their teams in gaining resilience. To support this, a study was conducted that aimed to answer the following research questions:

- Which specific situations are rated as “crises situations” by primary care practices?
- Which aspects need to be fulfilled in order to perceive a challenge as a crisis?
- Which strategies can be identified for managing these different situations successfully?

## Methods

### Study design

A qualitative observational study was conducted from June 2021 until February 2022. Ethical approval was obtained by the Ethics Committee of the Medical Faculty Heidelberg (S-456/2021). All participants gave their written informed consent prior to the interviews and focus groups. The study was documented in accordance to the Consolidated Criteria for Reporting Qualitative Studies (COREQ) checklist [12].

### Study sample

A purposive sample of a maximum of 40 experts was planned. As experts, stakeholders of primary care practices in Germany, such as physicians, medical assistants, practice managers, quality managers, hygiene managers were asked to participate in this study. Furthermore, experts from institutions on health system level, such as policy, research and health insurance focusing on primary care practices, were addressed. All participants needed to be 18 years and older and able to give consent. They were recruited within the personal networks of the institutions involved in the conduction of the RESILARE project such as networks of the project advisory board and via newsletter of the aQua Institute for Applied Quality Improvement and Research in Health Care GmbH. Additional snowball sampling was applied. The sampling strategy aimed to reach a variety in the geographical location of practices within Germany (north/south/west/east and urban/ rural), number of practice staff, different forms of practice organisation (single practices, joint practices, medical care centres, networks), medical disciplines and in the specific profession of the experts.

Staff of primary care practices in Germany were asked personally, by mail or phone to participate in either focus groups or interviews. If participants were affiliated to the same network or institution, they were asked to participate in a focus group. With all other experts, single telephone interviews were conducted.

Within the recruitment process, the thematic focus of the interview was headed with “crisis resilience”. However, some of the participants knew in advance that climate change might be a sub-topic of the interview/ focus group because they were recruited by members of the project advisory board who were engaged with climate change issues in healthcare. In addition, the project website mentions that one of the secondary target criteria of the RESILARE-project is to identify starting points and measures to reduce the ecological footprint of ambulatory medical practices.

### Data collection and measures

Data were collected in interviews and focus groups using a self-developed semi-structured interview guide. For focus groups, the guide was slightly adapted to the setting by rephrasing questions to address multiple participants at the same time. The interview guide was also slightly adapted for participants who were not working within the setting of a healthcare practice at the time (researchers or politicians).

In a first step, the development of the semi-structured interview guide was based on a literature research on (organisational) resilience in health care. To search for evidence on resilience, the definition of Blanchet et al. 2017 [13] was used, who defined resilience of health systems “as the capacity of a health system to absorb, adapt and transform when exposed to a shock and still retain control over its structure and functions. Thus, health systems are resilient if they exhibit absorptive, adaptive or transformational capacity in the face of shocks of different intensity”. In a second step, the first draft of the interview guideline was evolved step-by-step by a group of experts (authors as well as further researchers of the University Hospital Heidelberg) with expertise in health services research, qualitative research, work experience in practices, quality management in practices, climate change and health, as well as a researcher who focused on the coping of German general practices with the Covid-19 pandemic. Table 1 gives an overview of subjects and subthemes that were addressed in interviews and focus groups.

**Table 1:** Overview of interview and focus group guideline topics

Subject	Subthemes
Organizational resilience in primary care practices	- Starting points, criteria for medical practices, opportunities, challenges, aspirations - Experiences in previous crisis situations, disaster management - Previous strategies, action plans, resources - What can be transferred from previous experiences for preparing medical practices for future crisis situations?
Resilience of primary care practices to climate change challenges	- Challenges in the context of climate change for primary care practices (current and future) - Meeting these challenges in the practice: possibilities, barriers, opportunities and risks, concrete starting points and strategies - Assessment of own role and attitude, attitude of experts in relation to the adaptation of the ambulatory health sector to health consequences of climate change
Reduction of the ecological footprint of the ambulatory health sector	-Assessment of own role, attitude and attitude of the experts -Barriers and facilitating factors in the implementation of measures to reduce the ecological footprint of primary care practices -Change of care processes and structures with regard to their climate friendliness

Data collection was conducted from July until October 2021, either as telephone interviews or online focus groups using an online meeting program of the University of Heidelberg (heiCONF). Participants of interviews and focus groups were included upon reception according to the entrance of the provided form which confirmed their willingness to participate in an interview or focus group discussion. The interviews were performed by a female researcher and doctoral candidate with approximately four years of experience in qualitative research in the Department of General Practice and Health Services Research at Heidelberg University Hospital (NL) and a female masters-candidate and trainee in the RESILARE-project (VF). Both, NL and VF, have a background in speech and language therapy, interprofessional health care as well as health services research and implementation science, and are both around 30 years of age. Supervision in conduction of the interviews was provided by MW, JS and AW, as well as experienced teachers of the masters-program, with interdisciplinary backgrounds for example in sociology, medicine and medical process management.

Some of the interviewees were recruited throughout the professional networks of NL and JS and were therefore personally known by NL in a professional context. As the development of the interview guideline was done only by NL and experts of the Department of General Practice and Health Services Research at Heidelberg University Hospital, the interview partners did not get any insight into the questions in advance of the interviews. Data collection was part of the very first phase of RESILARE. Project partners participating in an interview or focus group were therefore not yet informed about specific research questions and purposes of the data collection.

All interviews and focus groups were audio-recorded and hand-written notes were taken. The online focus groups were video-recorded as well to ease the transcription process and capture group dynamics (e.g., non-verbal agreement or disagreement such as nodding) properly. No interview or focus group was repeated and no transcript was returned to the participants for correction. All interviews and focus groups were transcribed verbatim. Within the focus groups, non-verbal communication was transcribed as well.

Prior to the qualitative data collection, all participants were handed a short questionnaire referring to sociodemographic data such as sex, age, profession and current working status, geographical location, number of practice staff and specific practice discipline (if working in a practice) as well as their level of education.

### Data analysis

For the qualitative content analysis, the pseudonymized data were first processed by randomly comparing them with the audio recordings and checking their accuracy. Next, an initial and pragmatic data analysis was conducted by NL in accordance with the Rapid Qualitative Analysis [14]. The analysis was done immediately after the individual data collection, based on the hand-written notes of the researchers. this facilitated a very quick proceeding without the necessity to wait for finalisation of transcripts. This initial analysis enabled a first understanding of the data and first categories and relevant aspects became visible.

In a second step, an inductive thematic analysis was performed on the full transcripts by NL following Thematic Analysis of Brown and Clarke [15]. The resulting codes were then synchronized with the initial categories which emerged from the rapid analysis.

Several methodological strategies were applied to ensure the trustworthiness of the analysis and findings. These include engaging with other researchers to minimize research bias, thus reducing the risk of losing relevant content. This was realized by accompanying presentations and discussions within the research team periodically during and after the coding process.

For data coding, MAXQDA software Version 20 (Verbi GmbH) was used, IBM SPSS Statistics Version 26 was used for analysis of sociodemographic data.

## Results

Two focus groups with 14 participants and 26 individual interviews with a total of 40 experts were conducted between 12^th^ July 2021 and 14^th^ October 2021. Because of the additional snowball recruitment, the participation rate cannot be calculated precisely but is rated as high, as all except for two directly contacted experts agreed to participate in the study. The additional snowball sampling provided more medical assistants with interest in participation than could be included in the study. Duration of the interviews varied between 39 and 94 min, with a mean duration of 55 min. Duration of the two focus groups was 80 and 91 min.

The study population included 60% female experts and most participants were between 40–59 years of age (42.5%). Most of the participants were working in a primary care practice, 14 were physicians, 16 were medical assistants of which 69% had an additional training as care assistants, practice managers, quality managers or similar. Few participants were working as researchers or in politics. Further professions included social workers, pharmacists and other health professionals. Table 2 provides an overview on sociodemographic data.

**Table 2:** Sociodemographic data of the study population

	**N**	**%**
**Total**	**40**	**100**
Sex		
male	16	40.0
female	24	60.0
Age		
18 – 24 years	1	2.5
25 – 39 years	14	35.0
40 – 59 years	17	42.5
60 years or older	8	20.0
Professional activity (multiple answers allowed)		
Working in a primary care practice as:		
physician	14	35.0
medical assistant	16	40.0
with additional training	11	68.8
other	3	7.5
Working in health system		
Research	3	7.5
Health system (Politics/health insurance/…)	4	10.0
others	9	22.5

The participants were working in disciplines of practices that are part of ambulatory care, including general practice, internal medicine, neurosurgery, pneumology, dermatology, orthopaedics, otorhinolaryngology and gynaecology. Practice sizes varied between single practices of two employees up to joint practices and practice networks with 40 employees. Participants originated from rural and urban regions all over Germany.

### Which situations are perceived as crises by primary care practices?

In total, participants described more than 60 different situations as crises for practices. Those situations can be divided into three domains: a) internal crises in practices, b) crises in health systems and c) overarching crises. Table 3 presents the categories of situations.

**Table 3:** Situations that are perceived as crises by primary care teams and selected representative citations

**a) Internal crises in practices**	
Breakdown of technical infrastructure	For example, caused by a blackout, technical devices like computer or telephone are out of order. Furthermore, failure of single technical devices such as the insurance card reader or a software used for electronic health record can cause a crisis for a practice. In this context a virus or hacker attack was named as well *“[…] if this stupid card reader doesn’t work right now, then I don’t know what to do […] And of course that’s another crisis.” (SP1_Int22)*
Disputes with patients or within the team	This ranges from patients that show dissatisfaction or verbal complaints up to offences, abuse and even violence against the practice staff. Aggression of patients was described to be an increasing problem in practices. Besides this, lawsuits, medical errors and negative ratings of practices on the internet that cause patients to choose a different practice for treatment are named as crises Furthermore, personal differences may occur within the team and can lead to practice split-ups in the worst case *“But you’ve also been spat at in the practice. […] Yes, well, there is also this kind of patients who don’t accept things, who then become really abusive and insulting.” (SP1_Int14)*
Damage to the building	Water damage, burglary or a damage/dysfunction of an elevator implying barriers of reachability for patients with walking disabilities can be seen as an internal crisis *“water damage in endoscopy, yes, that’s a crisis.“ (SP1_FG2)*
Medical emergency	Medical emergency situations on patient level, like a heart attack or a stroke were mentioned. This was primarily named by practice staff that announced a lack of knowledge in handling these specific situations *„Reanimation, resuscitation, in other words, life-threatening emergencies, […] Let’s summarize it like this. That is literally also a crisis.” (SP1_Int23)*
Inspections	Some participants described a visit for inspection, e.g. hygiene inspections conducted by a health department or similar, to be a crisis for them as these visits cause a high workload in advance and may bring organisational consequences for the practice when deficiencies are being identified *“[…] but it actually also fits a crisis: the announced visit of the health department to check the practice.” (SP1_Int2)*
Staff shortage (temporary)	Temporary staff shortage may be caused by acute illness of staff, pregnancy and maternity leave or longer lasting illness. Some participants even described situations as a crisis that are actually not extraordinary, just because of a lack of staff to cope with it *“[…] a few years ago quite a lot of medical assistants became pregnant [laughs] five at once, […] and it is generally, if important employees in each level suddenly leave the unit – that does not have to be a big crisis, but it can become one.” (SP1_FG2)*
**b) Crises on health system level**	
Staff shortage (long-term)	Most of the participants mentioned a long-term and increasing staff shortage in medical professions as serious crisis for practices and on health system level. As crises on practice level, retirement of physicians resulting in open job offers and closure of the practice if no replacement can be found was named. This was described to be resulting in a shortage of practices, especially in rural regions, resulting in a higher workload for existing practices. Working conditions were described to be increasingly unattractive. Therefore, participants stated that especially younger staff would prefer to work in joint practices with a good infrastructure. Furthermore, participants mentioned that it is becoming increasingly difficult for them to find well-trained staff. This was specifically named for medical assistants Staff shortage not only occurs in the practice itself, but was also named to be relevant for nursing homes and ambulance service. Participants perceived that their own workload increases due to a lack of this external staff. This was named primarily by staff of general practices as they have to compensate staff shortage in nursing homes by a higher number of visits *“Well, lack of personnel in the first place. Yes, I see it as quite a big problem everywhere. (SP1_Int_09)*
Supply shortage	Participants named a shortage of vaccines (influenza, covid-19 and other), medication and medical devices as a periodically reoccurring crisis for practices. Especially in the context of the first phase of the covid-19 pandemic, a massive shortage of face masks, disinfectants and other protective equipment was named *“The first major crisis is always supply bottlenecks. We have seen this quite a few times for example with influenza [vaccines] or other important drugs.” (SP1_Int_05)*
Increasing care needs	Care needs are described as increasing steadily and are predicted to keep increasing in the future. This was mentioned in the context of demographic change, an increase in chronically ill and geriatric patients, as well as an increase in patients with mental illness that tend to require a higher need for consultation. Along with this, participants described the increasing care needs to become a crisis especially in the context of increasing staff shortage Besides these long-term developments, an acute disaster affecting many persons at the same time was also described as possible crisis for practices as they cannot cover to treat an extremely high number of patients
Changes in health system infrastructure	As changes in the infrastructure of the health system, centralization of health facilities and local relocations were named. Because of these, specific areas might be perceiving a shortage of care facilities (especially in rural areas and districts with high poverty). Few participants described that for example a practice in their neighbourhood decided to discontinue home visits as they bring no financial benefit to the practice. This led to the own practice having to additionally care for these patients by making home visits *“[…] there are of course also, let’s say, structural crises at the local level. Doctors joining forces or pharmacies getting bigger, retail or, let’s say, frequency structures changing, a large medical centre being built somewhere, the clinic spreading out into outpatient care in some form or other, that can of course also be difficult.” (SP1_Int5)*
Digitalization	Digitalization was named as crisis for practices on three different levels. First, participants perceived the transformation itself as a crisis when their technical affinity was described as low. Some mentioned that especially older physicians and medical assistants refused to deal with and implement technical approaches in order to “*sit this one out*” until they retire. Second, technical affinity was also described as low in some older, chronically ill patients who were said to “*get left behind*” by the digital transformation process in healthcare. Third, digitalization was named as a crisis whenever the technical devices implemented in the practice failed (see “Breakdown of technical infrastructure”). One medical assistant expressed concern about being replaced by machines in the future *“If you like, this is an approach to solving crises – but the path until digitization is properly implemented can still be a crisis.” (SP1_Int6)*
Social crises	Social crises in general could also affect primary practices. In particular, migration and the care of refugees were named as crises for practices as they perceived a high workload. Along with this, participants named that they had to treat diseases that they have never been confronted with yet, which resulted in a crisis for them *“[Another participant from focus group] mentioned the refugee crisis, because we were very much involved in the care. Partly communication was not possible, I think that some colleagues were also quite afraid when they had to go to the refugee accommodations. So, I think there were actually different things that felt like a crisis […].” (SP1_FG02)*
**c) Overarching crises**	
Epidemic/pandemic	For most participants, the current pandemic of covid-19 was the first and most significant crisis that came to their mind. Besides covid-19, Ebola, H1N1, influenza, gastrointestinal diseases and local outbreaks of paediatric diseases (e.g. in schools or day care) were named. Most of the participants expect further disease outbreaks like the covid-19 pandemic or other, new viruses in the future *„I think that through the climate crisis, […] through the pandemic as a whole, so a lot of things in medicine will change as well.” (SP1_FG1)*
Economic crises	As economic crisis on health system level, a shortage or shift in the payment of health care was feared. Due to social insurances, funding might lack with increasing poverty and unemployment. Besides this, participants concerned that they had to cope with the increasing care needs but will perceive payment cuts at the same time which might lead to redundancies of practice staff. Furthermore, concerns about financial losses due to a predicted decrease of treatments that require out-of-pocket-payment (IGeL), or due to restrictions of funding were described (increasing care needs and decreasing funding rates at the same time) *“An economic crisis may occur.” (SP1_Int5)*
Local disasters	Local disasters such as damage in a nuclear power station or a fire of industrial companies located in the neighbourhood of the practice were named as possible occurring crisis situations *“[…] these fears, well, for example nuclear power plants – we have one in 60 km distance – what else is going to happen? Can this also erupt like Chernobyl? (SP1_Int1)*
Climate change	Some participants already named climate change as an upcoming crisis by themselves and few were even using the term “climate crisis” instead of “climate change”. Some saw consequences of climate change but did not perceive them to be a crisis and a few did not see any consequences for practices at all as they haven’t yet thought about possible impacts of climate change. But generally, climate change was associated with effects on practices on many levels. In this context, heatwaves were mentioned primarily. Many participants already perceived periods of extremely high temperature in their practices. Described consequences were: patients that could not come into their practice during that time, damage on medication that was stored in a badly ventilated room, dehydration or bad health condition of patients and staff, worse health outcomes of patients after (ambulatory) surgery and a slow recovery after sedation, failure of medical technique such as ultrasound, higher workload due to extra home visits and visits of nursing homes with patients suffering from heat-related illnesses, up to the need for an acute shutdown of the practice. Besides heat waves, other extreme weather events such as floods, storms, cold spells, heavy rain or snow, black ice were named as possible consequences of the climate change. Those extreme changes of weather were predicted to increase symptoms of migraine, back pain, gout and arthrosis (weather changes), asthma and COPD (higher humidity) and longer and more intense allergy seasons. An increase of mental illness was named in the context of climate change as well. Only few participants named the occurrence of tropical diseases, but many named an increase of vector-borne diseases and saw a link between new occurring viruses like covid-19 and climate change. A general change in the range of diseases because of changing environment was prognosed. This was also named in the context of forced migration due to climate change. As further consequences of climate change, a shortage in resources such as water, nutrition and power were mentioned *„Climate change or the climate crisis will certainly have an impact on practices.“ (SP1_Int2)*

### Which aspects need to be fulfilled in order to perceive a challenge as a crisis?

Most participants perceived that the rating of a situation as a crisis is very subjective and depends on the characteristics of the individual experiencing the specific situation. However, the following three main aspects could be identified as required for rating a situation as a crisis: a) emotional reaction, b) organizational changes and c) lack of resources.

- Emotional reaction: A crisis was described as mental stress that sometimes inhibits a rational reaction. In the eyes of the participants, a crisis implied a risk for a burn-out and personal limits being exceeded. Discussed specific emotional reactions included: fear, stress, desperation, the feeling of losing control, insecurity, helplessness, overload and kind of shock-induced paralysis. In context of the pandemic of Covid-19, some participants described a fear of death. The level of emotional response differed within the participants and the respective crisis situation, but all participants described mental stress regardless of the severity of the crisis situation.
- Organizational changes: In all cases, a crisis situation was described by a change of routine and requiring different kinds of reorganization and change. Participants perceived that processes in practices needed to be adapted to the changing environment. Some underlined that a crisis also offers a chance for developing and improving practice processes and explicitly mentioned this as a positive aspect of crises. Processes in practices not only were seen in need to be adapted, but were described as being inhibited by the crisis. It was mentioned that previous routines became disturbed and could not be continued as planned. Some mentioned a certain chaos that occurred. One participant stated that existing vulnerabilities and weaknesses in practices are highlighted throughout a crisis situation.

*“When crises come, no matter in what form, weaknesses always reveal themselves everywhere, which were actually already visible for a long time theoretically and (…) were fallow. That actually nothing was ever done against it, and, if then such a crisis comes, like the pandemic for example, then such a thing becomes quite often to the disadvantage, I noticed.” (Int. 15, medical assistant)*

c) Lack of resources: In general, crises were perceived to mean a high “*working load exceeding the normal level*” (Int. 4, physician), resulting in an overload of available resources such as staff, time, material, knowledge or money. Especially a lack of practice staff was rated as a significant aspect of a crisis. Some participants described that a certain crisis situation might not have been named a crisis if they would have had enough staff to cope with it. A lack of medical supplies such as vaccines, medication or medical devices was described as turning a regular situation into a crisis, because the lack of supplies makes it impossible for the practice staff to respond to the specific situation properly. A crisis situation was also reported to be always linked with a lack of time while requiring a timely reaction.

“*Yes, the time pressure is certainly one of the central characteristics of crises, definitely!*” (Focus Group 1, Part. 1, physician)

Participants also described crises to appear suddenly or building up slowly but escalating quickly. The crisis situation itself was mostly described to be a longer lasting situation. Besides the necessary timely reaction, a crisis was also described by some participants to be linked with economic losses for the practice, meaning an existential threat in the worst case. Knowledge about the specific situation was named to be another essentially required resource, since a knowledge deficiency was described as directly linked to emotional reactions such as helplessness and insecurity. If participants did not know how to cope with and respond to the occurring challenge, it was perceived as a crisis. Furthermore, a crisis was described as a new and unknown situation for which practice staff was not able to prepare themselves. In connection with this, crises were described to be obscure in their progression and impact and that therefore often there was “*no end in sight*”.

### Strategies to enhance resilience of practices

The awareness of the occurrence of a specific crisis situation and the practices’ willingness to prepare for it, seemed to depend on their individual degree of affection. If only other practices (even in their direct neighbourhood) were affected by a certain situation, some participants described that they felt lucky they were spared and got out of the situation without any consequences. Furthermore, the willingness to learn from past crises varied. Some participants described they developed action plans within past crises to avoid the same unstructured procedures in future occurrences of the same or similar situations and described this to have been helpful during the Covid-19 pandemic. Others expressed their anger as they felt their practice manager refused to learn from past crises such as H1N1. They described that the German health system was in a somewhat luxury situation as there had always been enough resources to cope with crises. In this context it was described that past crises were not “*serious and exhausting enough to learn from it*” (Int. 12, expert of institution on health system level). The assumption that crisis prevention primarily needs to be an investment requiring money, time and effort and does not bring any immediate effects, was described as discouraging for practice managers to invest in prevention measures. The drawn conclusion was that, if a practice was prepared well for a certain crisis, the specific situation would not be perceived as a crisis when it occurred. Therefore, some participants were concerned that managers might not see a benefit of previous investment in crisis prevention when assuming the situation had not been that bad after all. This concern was explicitly described as being based on personal observations. Most participants showed a high tension for change as they were currently experiencing the Covid-19 pandemic as a crisis. In this context, the project RESILARE was considered an opportunity to increase resilience of practices and that awareness would increase throughout the conduction of the project.

In general, participants expressed a need for concrete and feasible action plans. The following strategies were mentioned either as an experienced coping strategy from past or current crises or as strategies that participants felt should be implemented for future crises in order to successfully deal with these. A number of measures were considered helpful to build up preparedness of practices which was seen as another aspect of resilience in terms of crisis prevention. The resilience of individuals in practices was described as highly relevant and seemed to build a basis for the organizational resilience of the practice. Besides the support of the resilience of every individual, communication and team work were described as central measures to improve resilience of practices. Supporting individual resilience and team work was described as a foundation for a resilient practice and basic values on which in the following, specific procedures and coping strategies might build up on. The identified strategies were subdivided into four domains: a) crisis prevention, b) individual resilience, c) team work, and d) practice procedures / responding to a crisis. As the strategies were consistent, a summary is presented in Table 4.

**Table 4:** Strategies of primary care teams for management of crises and selected representative citations

**a) Crisis prevention**	
Building awareness	B being informed about what might occur in the individual practice and be connected with warning systems such as local warning apps was named to increase awareness. Also, participating at different trainings about diseases, climate change impacts, or specifications (like care assistants or study nurses) were named as possible approaches *“In my opinion it is […] just as [other focus group participant] has already said: the practice must develop further and […] that we have to begin to develop strategies for ourselves so that we do not come unprepared in similar situations.” (SP1_FG_1)*
Gaining knowledge	Together with increasing awareness, gaining specific information about possible crisis situations and transmitting this knowledge to all team members was named as way to prepare for a crisis *“So, I think what has definitely helped us every time and also works is […] an early recognition and a sensitivity, there is something right now or there is something coming up, that could become of interest.” (SP1_FG_2)*
Planning scenarios	It was recommended that all practices define possible upcoming crises and plan different scenarios that might occur. For each scenario, a concrete action plan should be prepared. Some participants described to rehearse those scenarios and action plans to feel safe and evaluate feasibility of the action plans *“Good preparation. > laughs < Prophylaxis is everything, prevention is very important – anticipating as well – ideally, anticipate what could happen, and then be prepared for it.” (SP1_Int_23)*
Providing resources (staff)	Providing an adequate number of staff was seen as one of the most relevant aspects of crisis prevention. For this, working conditions should be improved to keep fluctuation rates low and avoid open job offers. Supporting this, trainees and internships were mentioned as helpful and “cheap workforce”. Training all employees to be able to roughly manage other team positions can help if an acute replacement is needed. Also, a pool of staff that is shared with other practices or within a joint practice is seen as beneficial. For the participants it was important that external staff already knew the practice in advance to avoid initial training during a crisis *“And you’re not crisis-resistant if you […] don’t have enough qualified staff, aren’t you?” (SP1_Int_22)*
Providing resources (material)	It was recommended that a practice includes enough storage space, just in case something has to be stored within a crisis. On top of that, it was asked by some participants that all practices always have a back-up in their most used items such as gloves, face masks, disinfectant, frequently used medication and medical devices *“[…] we then have really procured this personal protective equipment ourselves […] in a manageable amount – we were not a huge practice, but rather a medium-sized practice – but that we had such a basic equipment of these materials, we have stored in the practice and could then fall back on it in a new case […].” (SP1_Int_23)*
Providing resources (financial)	As crises were often linked with a financial burden for the practice, providing financial security for a certain time with no income was named as an important coping strategy *“[…] that was a period of four weeks, then the bosses fortunately still had some financial reserves for us and our salary and then we could bridge that.” (SP1_Int_03)*
Quality management	The overall conduction of quality management in practices as their participation in quality circles was seen as one way to improve organizational resilience already *"Well, I think what can definitely help a practice in such situations is quality management.” (SP1_FG1)*
**b) Individual resilience**	
Satisfaction at work	Participants expressed their need for supporting their own mental health and satisfaction at work through a good and appreciative management, through inclusion of their mental health status and feelings in the communication within the practice team and, through creating a healthy working environment *“Well, I would tell the physicians: keep your team together. […] Have keen senses, ask how they are doing and take them on board, the medical assistants, because they are on the front line and they have to communicate and lead and organize. And I think that’s where a lot of people are stuck or there’s a lot of potential for errors or crisis potential.” (SP1_Int_17)*
Beneficial characteristics of individuals	As beneficial characteristics of individuals, the following personal qualities were named: creativity, flexibility, adaptability, openness, curiosity, personal commitment, working experience (in particular: knowing your patients for a long time), active confrontation with the crisis, seeing the crisis as a chance, staying and acting calm, keeping a distance to the crisis, self-protection. In this context, participants mentioned that a practice has to know and accept its limits: *„[…] and perhaps also to admit: we can’t accomplish everything. So (that you) have to admit to yourself as a practice, with our resources we can’t manage to vaccinate all the people who want it immediately. I1: Why do you think it is important to admit that? Int_2: Because otherwise it is a constant overload.“ (SP1_Int_2)*
Individual attitude towards crises	Especially younger medical staff was rated to be less resilient than older staff. Two participants based this on the assumption that those persons were raised differently, in a “softer” way than themselves. Additionally, it was described, that especially physicians were likely to see crises as something positive and even tend to be happy when a crisis occurs: *“[…] but then doctors, well (…) they also find it kind of chic, a bit of a crisis… then it finally tingles in the stomach again.” (SP1_Int_12)*
**c) Team work**	
Team meetings	As one of the most important strategies to cope with a crisis successfully, team meetings were named by all participants. Team meetings were described to be necessary in the regular patient care and needed to be held more frequently during a crisis (e.g. weekly or daily depending on how quickly a crisis situation is changing). To achieve a good team communication, it was seen as necessary to consider emotional aspects and the mental health state of the team members as well as the allocation of tasks and responsibilities during a crisis. Furthermore, all team members should have the same level of information about the crisis *"Certainly, communication within the team […]? It is clear, that the flow of information must be guaranteed, that there is clarity and that everyone is informed: what is the matter, what is the significance, what are the consequences and where do we have to set other priorities under certain circumstances?” (SP1_Int_23)*
Different levels of education	It was important for the participants to be aware of different levels of education within the team (physicians vs. medical assistants) and provide transparent and comprehensible information for all *“And what is also important, is that the employees come from different areas, for example there were some who are really close to the patients […], then also some from the administrative area […] a colorful mixture, so that everyone can really give his or her input.” (SP1_FG2)*
Atmosphere within the team	A constructive error management, diversity within the team (e.g. languages and nationalities, education level, specifications, age), and a good team atmosphere in general were also identified as beneficial. For a good team resilience, it was observed to be crucial to have a feeling of “moving in the same direction” (German “am selben Strang ziehen”) *“We need this wide range of people, we need young people, we need old people, and everyone has his or her right to exist. So, we also need a colleague who maybe knows another language […].” (SP1_Int_21)*
Leadership style	To support a beneficial team work, a good practice management with an officially trained manager was seen as crucial. Low hierarchies and delegation of tasks was welcomed by the participants but at the same time, the practice manager should not give the feeling of pulling himself back. If a conflict occurred within the team, the consultation of an external and neutral person was asked *Interviewer question: “Is there anything else you would say a medical practice needs to be more successful in dealing with a crisis?” Interviewee’s answer: “A good boss. > laughs < A good boss who really backs the team.” (SP1_Int_11)*
**d) Practice procedures**	
Detection of crises and information acquisition	First, early detection of the crisis situation and immediate analysis of the occurring problem were described. After this, gaining information about the specific situation or problem and always stay up to date with the changing environment were named. Additionally, it was important that all information was shared within the team *"Well, I think it just needs a lot more awareness and information […] what could happen to us, which we perhaps have not even considered yet." (SP1_FG_1)*
Action plan	Another important strategy was to use existing action plans and, if no action plan was present, create an individual action plan. Within these action plans, all relevant steps, tasks, responsibilities and, if necessary, contact information of relevant institutes or persons needed to be included. Furthermore, the respective action plan needed to be feasible for the realisation within the individual practice environment. To respond to a crisis, this respective action plan needed to be implemented step by step to achieve structured and sensible proceeding. Especially during the covid-19 pandemic, this was seen as difficult due to a lack of consistent information and hardly feasible action plans for German practices *“There are plans how we are to behave, if it comes somehow to pandemic symptoms. Exactly, this already exists now and has also been established in our practice. And yes, you can orientate a bit on that […] Well, that’s a manual […] where things are simply laid down how you should behave […].” (SP1_Int_09)*
Adaption to mental and physical health of staff	As the crises usually implied a higher workload for the practice team, spending overtime hours, cancelling vacation time, increasing working time of part-time staff, or giving staff a time out to protect their health were named as strategies. For this, it was seen as crucial to adapt the specific strategy to the mental and physical health of the individuals. Another strategy to support resilience of a practice was to provide periodically reflection/evaluation sessions with all team members. “What went well? What didn’t? And what needs to be changed for the next step?” were important questions, the teams were discussing. This can be linked to the team meetings and should be part of the error management *“[…] and also to recognize who is reaching his or her limits. We have a doctor […] who also reached her limits because she worked more, and she then got two days off in between. […] I think motivation is very important.”*
Pro-active approach and immediate action	In general, a pro-active approach and immediate action was seen as beneficial in responding to a crisis successfully. Some participants described that their practice managers have waited too long so that it was more difficult to respond to the crisis, others praised their practice managers if they were acting immediate and were able to catch up the situation or prevent certain problems that became visible in other, non-prepared practices (e.g. buying enough face masks and disinfectant during the covid-19 pandemic) *"[…] whereas what I think has really helped us a lot – regarding our basic attitude and our strategy – is that we have always tried to deal with these issues proactively." (SP1_FG2)*
Networking	Information exchange and networking, not only within the team, but also with other external institutions such as other practices, hospitals, health departments, political or funding institutes (e.g. health insurances, associations of statutory health insurance physicians), professional associations, local authorities, nursing homes, pharmacies, disaster control authorities, and similar are rated as crucial to build resilience. In this context, exchanging information and experiences with the implementation of coping strategies (f.e. via E-Mail, Whats-App, personal meetings, online meetings, quality circles) or the exchange of resources (like staff or medical devices) was described as helpful *“I think what is helpful in such ordinary everyday crises or also when it’s about business-threatening issues and so on, […] or also in the doctors’ network, is, yes, to reveal yourself to others, to talk about things, to ask for help, to ask others how they are doing. Have you ever had the same thing? I am in a certain situation, I can’t get out of it. Well, not to look for facts in the first place, but to identify where I can get support quickly and easily?” (SP1_FG1)*
Changes in practice procedures	First, prioritizing of tasks and patients’ needs was named as a possibility to allocate resources efficiently. Second, changes in managing patient flows included the separation of infectious patients from non-infectious patients (especially within the covid-19 pandemic) and implementing specific consultation hours just for potentially infectious patients was named by almost all participants. Together with this, the participants described that they have implemented the need for patients to call and make an appointment before coming into the practice. Most participants rated this change as highly beneficial and wanted to stay with this in the future. Some participants described that they implemented other, specific time slots within their practice like a time slot for processing prescriptions, slots for vaccination, and other. It was also of importance to not plan workflows too tight so that they will include enough time to deal with unexpected issues *"These are all very big and very urgent things that have to happen quickly, but we can’t react to everything, we have to set hierarchies, prioritizations. And, above all, we have to make sure that we can work as a team." (SP1_Int_02)*
Communication with patients	Communication with patients needed to be transparent, comprehensible and patient-friendly. It was seen as crucial to provide all information to patients to make them understand certain changes in care provision and catch up their fears and needs adequately. Along with this, management of complaints and periodical patient surveys were named as beneficial. Furthermore, patient compliance was described higher when they were informed. As compliance was described to decrease within longer-lasting crises, communication needed to be “refreshed” periodically. For specific crisis situations that affected certain patient groups (like heat waves), it was necessary to inform those vulnerable groups about the occurring crisis and coping strategies. The following concrete communication strategies were named within the interviews: a homepage with highlighted news and a contact form, contact opportunity via e-mail, a specific telephone hotline, information brochures/flyers, signs, information provided on social media (e.g. Facebook page of the practice) or an action sheet especially for patients *„Well, the more we communicated, the better it worked out, if I’m honest.“ (SP1_Int_05)*

## Discussion

The interviewed healthcare providers mentioned a wide range of crises, varying from situations in their practice organization and problems in the healthcare system to societal and natural disasters. Crises were seen as being characterized by emotional responses, need for organizational adaptations, and lack of resources. Discussed strategies to manage crises related to crises prevention, individual resilience of healthcare workers, team work, and procedures used in practice. These insights from people with lived experience can be used to inform policies and programs for the prevention and management of health crises at national and international level.

The breadth of the resulting catalog of (potential) crisis situations for primary care practices shows that the demarcation between actual crises, general challenges and other developments in healthcare that might be perceived as crises (e.g. digitization) is not always highly selective. The individual attitude of the respondents and the ability to adapt quickly to unfamiliar situations or short-term changes obviously plays a central role in this classification. This became particularly evident when discussing the topic of climate change, where a wide range of attitudes came to light. While some respondents equated climate change with climate crisis, others did not feel any consequences and therefore did not see any challenges with regard to this topic. This phenomenon also was described by Van Lange and Huckelba (2021), who found that the topic of climate change is much more present when one experiences it oneself or is directly affected [16]. They therefore stated that possible solutions for climate change should also be addressed on a microlevel in order to make it more tangible [16]. Regarding the mentioned crisis situations, it is remarkable that bioterrorism was not mentioned by the interviewees. This topic was discussed especially in the US [17, 18] but does not seem to play a crucial role in the context of German primary care institutions. The same applies to the mentioning of war as a possible crisis. However, it should be noted here that the interviews and focus groups were conducted before the outbreak of the war in Ukraine in February 2022.

When it comes to the mentioned strategies for handling challenges or crises, a lot of emphasis was put on the importance of prevention. Literature shows that a comprehensive and thoughtful prevention and preparation can contribute to averting certain crises for the practice [2, 19]. This is consistent with the findings of this present study as participants concluded that if one prepares specifically, for instance in the framework of a project like RESILARE, then certain situations will no longer be assessed as a crisis in the future. In the long run, this could lead to a relief for the outpatient health care system. Also, in the Covid-19 pandemic, the factor of individual preparedness of primary care practices in terms of availability of medical supplies, was apparent [20]. In line with this, Stengel et al. [11] recommend the development of concrete action plans for German primary care practices in order to be prepared for pandemics. Collins et al. [21] already called for adequate preparation for pandemic situations in their 2008 article. In particular, they emphasized the relevance of expanding human resources to deal with resulting challenges of a pandemic. In contrast to previous work, the interviews conducted here also focused on topics such as team communication and strengthening resilience of individual employees, which seem not to be comprehensively present in medical practices to date. However, from the interviewees’ perspective, empowering individuals and team employed in the practice can help to ultimately strengthen the crisis resilience of the entire organization [22]. Crisis resilience strategies published so far tend to be based on fixed behaviours related to specific situations. However, as the results of this study suggest, efforts should be made to leave room for flexible adjustments and thus be able to respond to different (potential) crisis situations.

In general, it should be noted that the respondents’ views on what constitutes a crisis and what is merely a general change in healthcare are subjective and the boundaries are blurred. Therefore, it is highly relevant that each practice individually considers which of the situations could become a crisis to their own practice and how to prepare for it. This provides a content-related reference to quality management in practices, as it aims to define quality targets, work out optimization approaches and identify concrete measures. In this respect, the approach of the RESILARE project to establish a link to the quality management of the practices and thus to promote a reflection of their own actions and practice organization referring to crises seems to be a promising one. Many aspects listed as possible crisis situations are addressed by quality indicators newly developed in the project to facilitate a stronger awareness of these aspects in the future.

### Strengths and limitations

Our study adds to a growing body of research on the concept of resilience and its importance in the context of healthcare. Until now, only a small part of this research has specifically highlighted the relevance of strengthening primary care practices in order to cope with different crises. The results of this study stress how many different dimensions, such as crisis prevention, individual resilience of team members as well as team work, and adaption of practice procedures, need to be addressed in order to enlarge resilience in primary care practice teams. By this it can contribute to strengthening primary care practices for future crises which is particularly important because they often are the first and most important point of contact for citizens needing help in emergency cases.

Despite the study’s focus on general crisis resilience, there is a possibility of sampling bias towards experts with a pronounced interest in the topic area of climate crisis and its impact in the health sector. Nevertheless, when selecting the study participants, maximum care was taken to ensure a balanced composition of the study sample to minimize the probability of the occurrence of a bias regarding the topic “climate change” by accounting for main work areas in the recruitment process. Still, it must be stated that participating experts were potentially better informed about climate change than others. This might also explain the high willingness to participate in the study. On the other hand, it can be noticed that interest in the topic has increased substantially among German ambulatory care providers in recent years.

Another limitation that has to be stated is that data collection took place during the Covid-19 pandemic and therefore the issue of pandemics might have been weighted more heavily than at other times. Nevertheless, the interviews revealed a very broad perspective that brought many different crisis situations to light.

In general, there was a very high need for discussion of the research topic on the part of the participants, particularly among medical assistants. On the one hand, this was reflected by a very high willingness to participate, which even made it necessary to select the participants, as too many came forward. On the other hand, this is also emphasized by the length of the interviews, which often exceeded the targeted 45-min duration.

For the data analysis it has to be stated that coding was executed by one member of the research team without double checking every transcript by another person. Nevertheless, the proceeding of the coding process was discussed conscientiously among the research team.

## Conclusion

The study provides insights into views on the topic of crises from the perspective of primary healthcare providers in Germany. In addition to the perception and classification of situations as crises, the study also focused on possible solutions and strategies for crisis management in the primary care sector. In particular, the relevance of strengthening communication within practice teams and resilience among employees was indicated as beneficial for the prevention of crises, or a better response to them. Adequate measures to achieve such strengthening need to be explored.

## Supplementary Information

**Additional file 1.** Guide for focus groups.

**Additional file 2.** Guide for interviews with other stakeholders.

**Additional file 3.** Guide for interviews with practice staff.

## Data Availability

The data supporting the findings of this study are not publicly available due to them containing information that could compromise research participant privacy.

## Abbreviations

COPD: Chronic obstructive pulmonary disease
COREQ: Consolidated Criteria for Reporting Qualitative Studies
Covid-19: Corona virus disease 2019
HIV: Human immunodeficiency virus
H1N1: Influenza-A-Virus H1N1, “swine flu”
IGeL: German “Individuelle Gesundheitsleistungen”, out-of-pocket-payment for health care treatments
Int.: Interview
