# Supplementary material for: Building resilience in German primary care practices: a qualitative study
Source: BMC Prim Care. 2022 Sep 2;23:221. doi: 10.1186/s12875-022-01834-4 (PMC9436723; doi:10.1186/s12875-022-01834-4)
Supplement: Supplementary file 2 — Additional file 2. Guide for interviews with other stakeholders. [file 12875_2022_1834_MOESM2_ESM.pdf]

RESILARE Study Phase 1

Guide for interviews with other stakeholders

Topic 1: Crisis resilience

1. What do you think would be examples of crises that might be faced by medical practices?
    a. What crises have you already witnessed in medical practices?
    b. What made the respective situation a crisis?
        i. What do you consider to be a crisis for medical practices?

2. What is your experience or impression of strategies that helped medical practices cope with crises?
        i. What strategies do you think will be helpful for medical practices in the future?
        ii. What is the role of existing guidelines? Which guidelines, and for what reason?
    b. In your experience, what strategies have not worked well in medical practices?
    c. What changes do you notice in medical practices after a crisis has been settled?

3. How would you generally assess the crisis resilience of medical practices?
    a. What do you think medical practices in general need to successfully deal with crises?
        i. In the area of training, competencies of employees in practice?
        ii. In the area of practice organization? (equipment, accessibility, staff)
        iii. In the area of patient information and counseling

4. When you look into the future, what crises do you envision? What other, perhaps new crises would you expect?

Topic 2: Climate change as a health crisis

5. Climate change has been identified in some studies as the greatest threat to global health in the 21st century. What might be in store for medical practices in Germany in this regard?
    a. How might this affect medical practices?
        i. … on the practice team?
        ii. … on the practice operation itself? (e.g.: Water becomes scarce during drought, power outage, extreme weather, heat in the practice, ...).
        iii. … on patients?

6. One of the possible consequences of climate change is the increase in heat waves in Germany, which also have health consequences.
    a. To what extent has this played a role in your practice to date?
    b. What advice do you give your patients?
    c. How do you rate the new guideline of the German College of General Practitioners and Family Physicians (DEGAM) in this context?

Mitigation

There is also a lot of discussion about what, for example, industry or each individual contributes to climate change. The keyword here is the ecological footprint. Measures are being discussed to reduce the individual ecological footprint, such as traveling less by airplane.

7. What do you think medical practices can do to help reduce the ecological footprint of healthcare?
    a. What measures aimed at such a reduction you may already be aware of?
        i. … in terms of material consumption in patient care?
        ii. … in terms of drugs or antibiotics?
        iii. … in terms of water and energy consumption in patient care?
        iv. … in terms of a healthy and climate-friendly lifestyle?

    b. What exactly do you think could be improved in this respect?
        i. … with regard to the measures themselves?
        ii. … with regard to the underlying conditions for the implementation of measures?
    c. What difficulties can arise in this respect?
    d. In your perception, how present are the topics of climate change and sustainability in German medical practices in general?

Conclusion/ Summary

8. One aim of the project RESILARE is to develop quality indicators to support medical practices in coping with crisis situations such as heat waves and waves of illness, which can, for example, be included in a checklist for medical practices. Thinking about the issues you just mentioned, what exactly do you think such indicators should take into account?

9. We discussed some aspects today, which ones have we perhaps not yet considered?
